# Signaling pathways mediating a selective induction of nitric oxide synthase II by tumor necrosis factor alpha in nerve growth factor-responsive cells

**DOI:** 10.1186/1742-2094-2-19

**Published:** 2005-09-06

**Authors:** Michael S Thomas, WenRu Zhang, Paivi M Jordan, H Uri Saragovi, Giulio Taglialatela

**Affiliations:** 1 Department of Neuroscience and Cell Biology, the University of Texas Medical Branch at Galveston, Texas - USA; 2 Department of Pharmacology and Therapeutics, McGill University, Montreal, QC, Canada

## Abstract

**Background:**

Inflammation and oxidative stress play a critical role in neurodegeneration associated with acute and chronic insults of the nervous system. Notably, affected neurons are often responsive to and dependent on trophic factors such as nerve growth factor (NGF). We previously showed in NGF-responsive PC12 cells that tumor necrosis factor alpha (TNFα) and NGF synergistically induce the expression of the free-radical producing enzyme inducible nitric oxide synthase (iNOS). We proposed that NGF-responsive neurons might be selectively exposed to iNOS-mediated oxidative damage as a consequence of elevated TNFα levels. With the aim of identifying possible therapeutic targets, in the present study we investigated the signaling pathways involved in NGF/TNFα-promoted iNOS induction.

**Methods:**

Western blotting, RT-PCR, transcription factor-specific reporter gene systems, mutant cells lacking the low affinity p75NTR NGF receptor and transfections of TNFα/NGF chimeric receptors were used to investigate signalling events associated with NGF/TNFα-promoted iNOS induction in PC12 cells.

**Results:**

Our results show that iNOS expression resulting from NGF/TNFα combined treatment can be elicited in PC12 cells. Mutant PC12 cells lacking p75NTR did not respond, suggesting that p75NTR is required to mediate iNOS expression. Furthermore, cells transfected with chimeric TNFα/NGF receptors demonstrated that the simultaneous presence of both p75NTR and TrkA signaling is necessary to synergize with TNFα to mediate iNOS expression. Lastly, our data show that NGF/TNFα-promoted iNOS induction requires activation of the transcription factor nuclear factor kappa B (NF-κB).

**Conclusion:**

Collectively, our *in vitro *model suggests that cells bearing both the high and low affinity NGF receptors may display increased sensitivity to TNFα in terms of iNOS expression and therefore be selectively at risk during acute (e.g. neurotrauma) or chronic (e.g. neurodegenerative diseases) conditions where high levels of pro-inflammatory cytokines in the nervous system occur pathologically. Our results also suggest that modulation of NFκB-promoted transcription of selective genes could serve as a potential therapeutic target to prevent neuroinflammation-induced neuronal damage.

## Background

Neuroinflammation is thought to play a prominent role in neurodegeneration associated with a variety of acute and chronic insults in both the central (CNS) and peripheral (PNS) nervous system [1,2]. Examples of neurotraumatic or neurodegenerative conditions where the occurrence or role of neuroinflammation has been documented include peripheral nerve injury [3-6], acute and chronic spinal cord injury [7-11], traumatic brain injury [12-14], stroke [15-17], amyotrophic lateral sclerosis (ALS, [18-20] and Alzheimer Disease (AD, [21-24].

Neurons susceptible to neuroinflammatory insults are often dependent for their survival on target derived neurotrophic factors such as nerve growth factor (NGF), brain-derived neurotrophic factor (BDNF) or glia-derived neurotrophic factor (GDNF). The same neurodegenerative conditions have also been associated with the presence of damaging high levels of free radical species leading to pathological oxidative stress [25]. For example, inflammatory involvement in AD pathogenesis has been proposed partly based on observations of increased levels of the pro-inflammatory cytokines tumor necrosis factor alpha (TNFα) and interleukin-1 beta (IL-1β) in cerebrospinal fluid and brain cortex of AD patients [26,27]. Additionally, among the most affected neurons in AD are the basal forebrain cholinergic neurons (BFCN, [28-30]), which rely upon trophic support by target-derived NGF [31,32]. Furthermore, there is strong evidence for the presence of oxidative damage in the AD brain [33-36]. Similarly, neuronal damage following acute spinal cord injury or peripheral nerve injury has been shown to involve a neuroinflammatory as well as oxidative stress component [1,8,10,11,37-39], and traumatic head injury is also known to be associated with increased circulating concentrations of inflammatory cytokines and reduced numbers of basal forebrain cholinergic neurons [13,40-42].

Thus, there seems to be an intimate relationship between pro-inflammatory cytokines, oxidative stress and trophic factors that underscores the neuropathological consequences of extrinsic (e.g. traumatic) or intrinsic (e.g. disease-related) injury to the nervous system. Our previous work has shown that in NGF-responsive rat pheochromocytoma (PC12) cells TNFα induces expression of the free radical nitric oxide (NO) synthesizing enzyme NOS II (iNOS) only in the presence of NGF acting through its high affinity receptor TrkA [43]. Indeed, perturbed levels of NOS and NO-derived oxidative damage have been reported in both acute and chronic neurodegenerative conditions [25], including spinal cord injury [44-46], stroke [47,48] and AD [49-53]. However, TNFα alone has not been shown to be an effective inducer of human iNOS promoter activity [54] or of rat cortical iNOS expression when administered intracerebroventricularly [55]. Nonetheless, TNFα has been shown to contribute to the death of NGF-dependent neurons *in vitro *[56] and *in vivo *[57,58]. Therefore, our previous results suggest the attractive idea that one mechanism through which increased levels of TNFα affect certain trophic factor-responsive neurons may involve NO-derived oxidative damage brought about by a synergistic induction of iNOS. Understanding the molecular mechanisms mediating the synergistic NGF/TNFα-promoted induction of iNOS may thus provide novel therapeutic targets for the prevention of certain neurodegenerative events associated with acute or chronic injury of the nervous system.

Here we report that a reversible expression of iNOS, produced in PC12 cells by simultaneous exposure to NGF and TNFα, requires the simultaneous presence of both the low-affinity p75NTR and the high-affinity TrkA NGF receptors. Furthermore, using specific inhibitors and a reporter gene assay, we show that such synergistic effect of the combined NGF/TNFα treatment is mediated by the transcription factor nuclear factor kappa B (NF-κB).

## Methods

### Materials

All routine reagents and chemicals were obtained from Sigma-Aldrich (St Louis, MO, USA), except where noted otherwise. Recombinant human and rat TNF and rat IGF were obtained from R&D Systems, Minneapolis, MN, USA, purified mouse NGF from Harlan Bioproducts, Indianapolis, IN, USA, and pyrrolidine dithiocarmbamate (PDTC), the octapeptide proteasome inhibitor (PSI), PD98059, K252a and 1400 W from Calbiochem, San Diego, CA, USA.

### Clonal cell lines

Stock cultures of rat pheochromocytoma cells (PC12; a kind gift of Dr. Lloyd Greene, Columbia University, New York, NY, USA) and PC12 cells lacking the low affinity p75NTR NGF receptor were maintained in 75 cm^2 ^tissue culture flasks in 10 ml RPMI-1640 culture medium supplemented with 5% heat inactivated fetal bovine serum in a humidified cell incubator at 37°C kept at a 5% CO_2 _atmosphere. Half of the medium was replaced every other day and the cells were split once a week to maintain cell viability.

### Expression vectors

Transient transfection of cells was performed by a liposomal packaging system. Briefly, 1.2 pmol of expression vector were mixed with DMRIE-C (Life Technologies, Carlsbad, CA, USA) in a 1:3 DNA to liposome ratio. The DNA/liposomes were diluted in 400 μl serum free transfection medium (Optimem) and then added to approximately 100,000 cells in a 12 well cell culture plate. The cells were allowed to take up the liposomal DNA for 3 hours before being washed and returned to cell culture medium. Cells were allowed to recover for 24 hours before any treatments. The cDNA coding for chimeric proteins bearing the extracellular domain of the TNFR1 receptor and the transmembrane and cytosolic domains of the NGF receptors (either p75NTR or TrkA) was a kind gift from Dr. Eric Shooter and prepared as described [77], (Stanford University, Palo Alto, Ca, USA). The p-SEAP expression vector, containing the SEAP gene under NF-kB, AP1 or CRE enhancer control, was purchased from Clontech (Palo Alto, CA, USA). Conditioned medium from cells transfected with the SEAP reporter vectors was assayed for alkaline phosphatase by sampling the medium and using the chemiluminescent Great EscAPe SEAP assay (Clontech, Palo Alto, CA, USA), according to manufacturer's instructions.

### Western blot analysis

Cells were lysed using an SDS-based lysis buffer (2% SDS, 5 mM EDTA, 50 mM Tris, 1 mM each of DTT, PMSF and protease inhibitor cocktail). Following an ice-cold PBS wash, cells were lysed with SDS lysis buffer and the sonicated briefly before clarifying by centrifugation at 20,000 g for 20 minutes at 4°C. After centrifugation the supernatant was collected and protein content was measured using the standard BCA protein assay (Pierce, Rockford, IL, USA). Protein extracts (40 μg) were diluted in 6X sample buffer and loaded onto a 6% SDS-polyacrylamide gel. Gels were run for one hour at 100 V and then were transferred to a nitrocellulose membrane overnight at 25 V. All incubations were at room temperature in 0.5% Tween in Tris buffered saline (TTBS). The membranes were blocked for one hour in 5% milk in TTBS. Primary monoclonal anti-iNOS (Signal Transduction Laboratories, San Diego, CA, USA) or polyclonal anti-TNFR1 (Santa Cruz Biotechnology, Santa Cruz, CA, USA) were diluted in 2.5% milk in TTBS at 1:1000 and membranes were incubated with the antibody for one hour at room temperature. Membranes were washed three times for ten minutes each in TTBS before incubating for one hour with a horseradish-peroxidase secondary antibody (BioRad, Hercules, CA, USA) at 1:7500 in 2.5% milk in TTBS. Finally, membranes were washed again in TTBS three times for ten minutes each. Immunoreactive bands were visualized by a chemiluminescent western blot detection kit (Amersham Biosciences, Piscatay, NJ, USA) according to manufacturer's instructions. Images were captured using a 12 bit monochrome camera (UVP, Upland, CA, USA).

### Reverse transcriptase polymerase chain reaction assay

Total RNA was extracted with Trizol Extraction Kit (Gibco BRL, San Diego, CA, USA) according to manufacturer's instructions. One μg of total RNA from each sample was applied to Ready-to-go RT-PCR Beads (Amersham Biosciences, Piscatay, NJ, USA) and used to complete the amplification protocol according to manufacturer's instructions. Primer sequences for rat iNOS were as follows; forward 5'-CAC GGA GAA CAG AGT TGG-3' and reverse 5'-GGA ACA CAG TAA TGG CCG ACC-3'. Amplified samples were run on agarose gels and stained with ethidium bromide. Images were captured using a 12 bit monochrome camera (UVP, Upland, CA, USA).

### Flow cytometry

One μg of antibody against TrkA or p75^NTR ^(Santa Cruz Biotechnology, Santa Cruz, CA, USA) was labeled with Zenon Rabbit IgG labeling kit from Molecular Probes (Eugene, OR) according to manufacturer's instructions and incubated for 1 hr with the cells in suspension. After incubation, labeled cells were visualized and quantified using a Becton Dickinson FACS Vantage Flow Cytometer set at appropriate instrument parameters.

### Statistical analysis

Where appropriate, data were expressed as mean +/- standard error of the mean (S.E.M.), and analyzed by student unpaired two-tailed *t *test with significance set at p < 0.05.

## Results

### Combined NGF and TNFα induce iNOS message and protein

**Figure 1:**
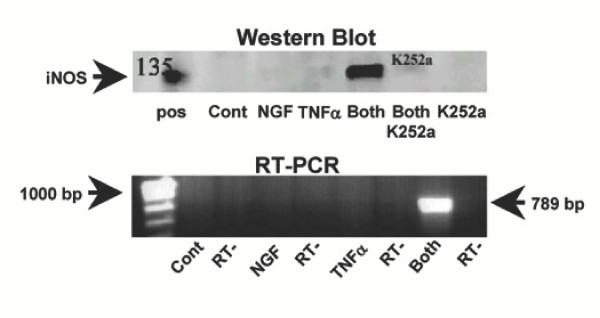
**A: (Top) **Western blot analysis detecting the presence of iNOS in 40 μg total protein extracts from PC12 cells treated for 24 hr with 10 ng/ml NGF and 10 ng/ml TNF, individually or combined (Both), in the presence of 50 nM of the receptor tyrosine kinase inhibitor K252a. Positive control (Pos) is 4 μg of total protein extracts from mouse macrophages. **(Bottom) **RT-PCR detecting iNOS mRNA in PC12 cells treated for 24 hr with 10 ng/ml NGF and 10 ng/ml TNF, individually or combined (Both) compared to untreated cells (Cont). Internal PCR controls lacking reverse transcriptase (RT-) were performed on each sample as shown. Results shown are representative of 3 replicate experiments.

The upper panel of figure 1 shows a western blot detecting iNOS in PC12 cells treated simultaneously with 10 ng/ml NGF and 10 ng/ml TNFα in the presence or absence of 50 nM K252a, an inhibitor of phosphorylative events associated with tyrosine kinase receptor activation that has been shown to block the function of the high affinity NGF receptor TrkA [61]. There was a marked induction of iNOS expression only in cells simultaneously treated with NGF and TNFα, while neither treatment alone elicited any effect. Furthermore, K252a completely abolished NGF/TNFα-promoted iNOS induction, suggesting that TrkA function is essential to mediate it. As shown in the lower panel of figure 1, along with increased protein levels there was also an induction of iNOS mRNA in PC12 cells treated with NGF and TNFα but not in cells treated with either factor alone.

### NGF and TNFα are both required for sustained iNOS expression

**Figure 2:**
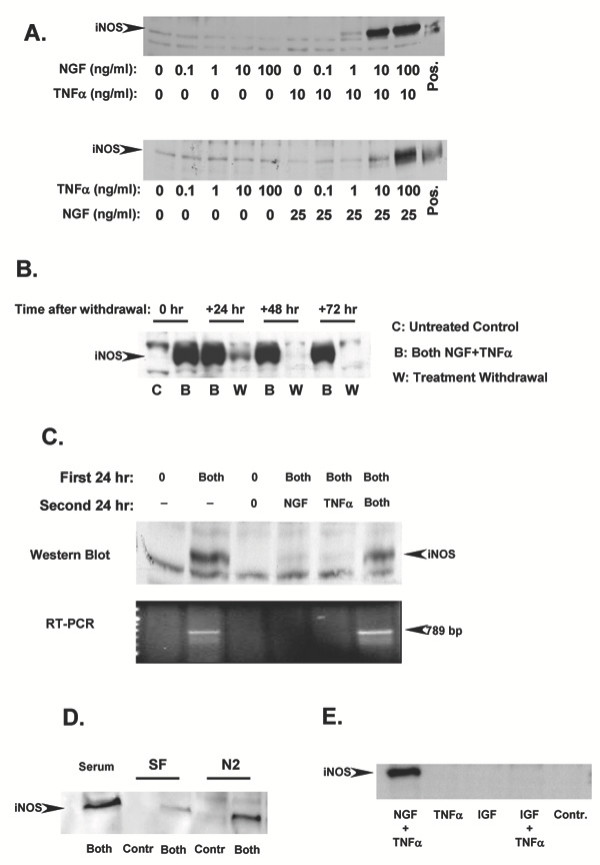
**A: **Western blots detecting iNOS in total protein extracts from PC12 cells treated for 24 hr with increasing concentrations of NGF in the presence or absence of 10 ng/ml TNFα **(Top) **or treated with increasing concentrations of TNFα in the presence or absence of 25 ng/ml NGF **(Bottom)**. Positive control (Pos) is 4 μg of total protein extracts from mouse macrophages. Results shown are representative of 2 replicate experiments. **B: **Western blot analysis detecting iNOS in total protein extracts from PC12 cells simultaneously pre-treated with 10 ng/ml NGF and 10 ng/ml TNFα. At 24 hr treatment was withdrawn and the presence of iNOS was determined 24, 48, and 72 hr thereafter. Results shown are representative of 3 replicate experiments. **C: **Western blot analysis **(Top) **and RT-PCR **(Bottom) **detecting iNOS protein and mRNA in total protein extracts and total RNA from PC12 cells simultaneously pre-treated for 24 hr with 10 ng/ml NGF and 10 ng/ml TNFα (Both). After 24 hr, treatment was withdrawn and replaced with either NGF or TNFα alone or with both and iNOS expression determined 24 hr thereafter. Results shown are representative of 2 replicate experiments. **D: **Western blot detecting iNOS in total protein extracts from PC12 cells simultaneously treated for 24 hr with 10 ng/ml NGF and 10 ng/ml TNFα in medium containing serum, in serum free medium (SF) or in defined medium (N2). Results shown are representative of 3 replicate experiments. **E: **Western blot analysis detecting the presence of iNOS in total protein extracts from PC12 cells treated for 72 hr with 100 ng/ml IGF and 10 ng/ml TNF, individually or combined, as compared to cells simultaneously treated with 10 ng/ml NGF and 10 ng/ml TNFα or untreated controls (Cont). Results shown are representative of 4 replicate experiments.

Figure 2A shows western blots detecting iNOS in cells treated with increasing concentrations of NGF (top panel) or TNFα (bottom panel), in the presence or absence of a fixed amount of TNFα or NGF, respectively. Either factor was ineffective when added alone at any of the concentrations tested. However, there was a marked dose-response increase in iNOS expression when increasing concentrations of NGF or TNFα were added in the presence of a fixed amount of TNFα or NGF, respectively. Figure 2B shows a representative western blot detecting iNOS expression in cells continuously treated with NGF and TNFα as compared to cells in which the combined treatment was withdrawn after 24 hr. The expression of iNOS returned to basal, undetectable, levels between 24 and 48 hr after withdrawal of both TNFα and NGF. Furthermore, as shown in figure 2C, withdrawal of either NGF or TNFα alone was sufficient to abolish iNOS expression induced by the combined treatment, both at the protein (top panel) and mRNA level (bottom panel). To exclude the involvement of unknown serum factors, NGF/TNFα-promoted induction of iNOS was determined in cells cultured for 24 hr in serum free or in defined medium N2 (Figure 2D). There was a detectable iNOS induction in both serum free- and defined medium-cultured cells, although much reduced in serum free conditions, which is predictable as PC12 cells do not survive for longer periods of time (24–48 hrs) in the absence of serum or N2 supplements. Since insulin is present in both serum and the N2 supplement, and can activate the insulin-like growth factor (IGF) receptor, we asked whether TNFα may synergize with IGF, which is also present in serum, to induce iNOS expression. The results shown in Figure 2E indicate that this is not the case.

### TNFα/NGF-mediated iNOS expression is independent of NOS enzymatic activity

**Figure 3:**
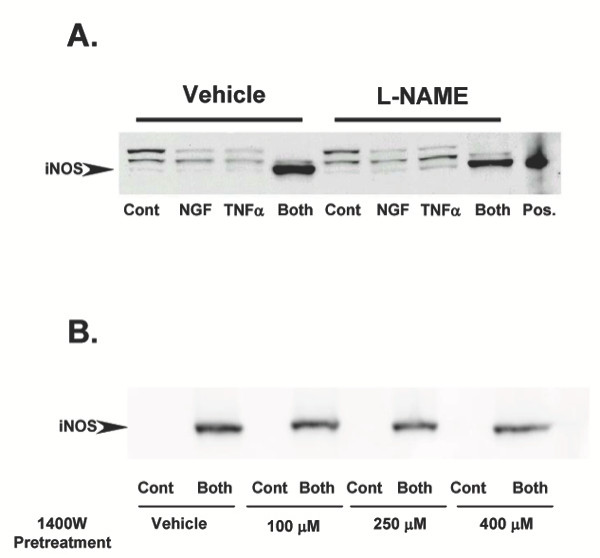
**A: **Western blot detecting iNOS in total protein extracts from PC12 cells treated for 24 hr with 10 ng/ml NGF and 10 ng/ml TNFα, either individually or simultaneously (Both). Cells were pretreated with vehicle or 0.5 μM of the generic NOS inhibitor L-NAME. Positive control (Pos) is 4 μg of total protein extracts from mouse macrophages. Results shown are representative of 3 replicate experiments. **B: **Western blot detecting iNOS in total protein extracts from PC12 cells simultaneously treated with 10 ng/ml NGF and 10 ng/ml TNFα (Both), in the presence or absence of a pre-treatment with varying concentrations of the iNOS-specific inhibitor 1400 W. Results shown are representative of 4 replicate experiments.

In order to determine whether the enzymatic activity of iNOS may play a role in sustaining TNFα/NGF-promoted signaling we pretreated PC12 cells with two NOS inhibitors prior to TNFα/NGF tretament. Pretreatment with N(G)-nitro-L-arginine methyl ester (L-NAME) did not affect expression of iNOS induced by the NGF/ TNFα combined treatment (Figure 3A). The same result was observed if a more specific inhibitor of iNOS (1400 W) was used instead of L-NAME (Figure 3B). Concentrations of 1400 W used here have been previously shown to be effective in inhibiting selectively iNOS activity in PC12 cells by others [78]. These results suggest that sustained iNOS expression in response of the combined NGF/TNFα treatment is independent of NOS enzymatic activity.

### NGF/TNFα promoted iNOS induction requires the transcription factor NF-κB

**Figure 4:**
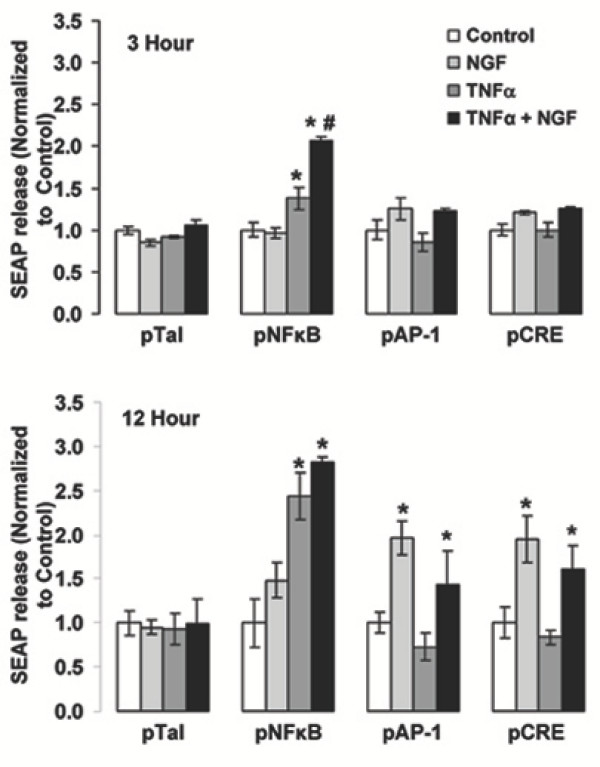
Detection of SEAP in the culture medium of PC12 cells transfected with a SEAP reporter gene construct under the transcriptional control of enhancers specific for NF-κB, AP-1 or CRE. pTal is the non-enhanced control SEAP reporter vector. Twenty-four hr after transfection, cells were treated with vehicle (Control), 10 ng/ml NGF, 10 ng/ml TNFα or NGF plus TNFα (Both) and the presence of SEAP in the culture medium assayed 3 hr **(Top) **or 12 hr **(Bottom) **thereafter. Results are normalized to control cells in each transfection group (N = 3). * and #: p < 0.05 vs. control and TNFα-alone, respectively (two-tailed unpaired Student's t-test). Results shown are representative of 3 replicate experiments.

Figure 4 shows results from PC12 cells transiently transfected with a secreted alkaline phosphatase reporter gene construct (SEAP) promoted by enhancer sequences specific for nuclear factor kappa B (NF-κB), activator protein 1 (AP-1), cAMP-responsive element (CRE) or Tal (non-inducible control). Twenty-four hr after transfection cells were treated with 10 ng/ml each of TNFα and NGF (alone or combined) and SEAP released in the culture medium (an index of endogenous transcription factor activation) was assayed 3 hr and 12 hr later. At 3 hr, cells treated with TNFα showed a significant increase in NF-κB activity but not AP-1 or CRE. Cells treated with NGF alone showed at 3 hr no significant increase in NF-κB, AP1 or CRE activity. When cells were exposed to the combined NGF/ TNFα treatment, there was a robust increase in NF-κB activity that was significantly higher than the response induced by the individual treatment with TNFα. On the other hand, neither AP-1 nor CRE activity were significantly affected by the combined NGF/ TNFα treatment. At 12 hr, both TNFα and NGF/TNFα combined treatments significantly increased NF-κB activity, but were not statistically significantly different. NGF-treated cells showed a significant increase in AP-1 and CRE activity at 12 hr, while NF-κB activity was not affected. As a result, there was also a significant increase in AP-1 and CRE activity elicited by the NGF/TNFα combined treatment at 12 hr. Neither NGF nor TNFα (alone or combined) elicited any effect on the control reporter construct Tal, either at 3 or 12 hr.

**Figure 5:**
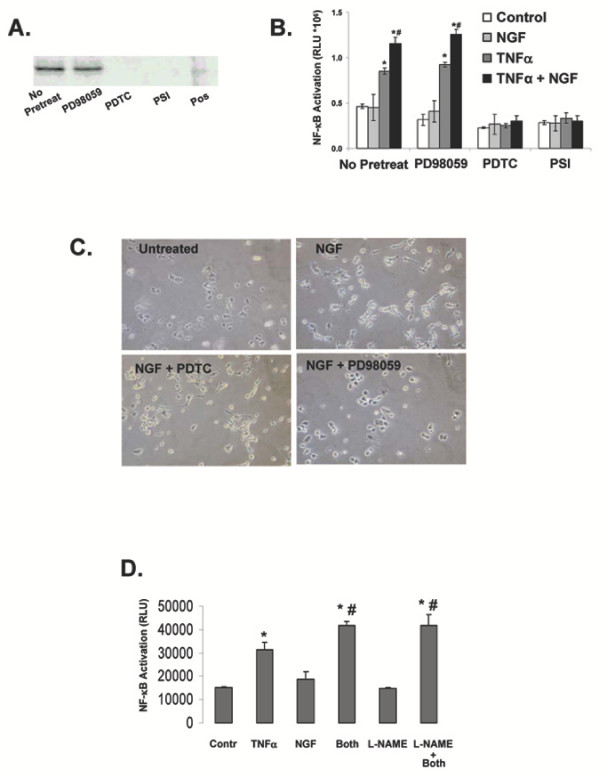
**A: **Western blot detecting iNOS in PC12 cells simultaneously treated with 10 ng/ml NGF and 10 ng/ml TNFα for 24 hr. Thirty minutes before NGF/ TNFα treatment cells were pre-treated with 10 μM pyrrolidinedithyocarbamate (PDTC), 2 μM of a oligopeptide proteosome inhibitor (PSI) or 10 μM of a MAPK inhibitor (PD98059). Results shown are representative of 2 replicate experiments. **B: **SEAP release in the culture medium of PC12 cells transfected for 24 hr with an NF-κB-sensitive SEAP reporter gene construct and treated for 12 hr with vehicle (Control), 10 ng/ml NGF, 10 ng/ml TNFα or NGF plus TNFα in the presence of 10 μM PD98059, 10 μM PDTC or 2 μM PSI. Data are shown as mean ± S.E.M. from 3 independent replicate experiments. * and #: p < 0.05 vs. control or TNFα-alone cells, respectively (two-tailed unpaired Student's t-test). **C: **Representative photomicrographs of PC12 cells treated for 48 hr with 10 ng/ml NGF in the presence or absence of 10 μM PD98059 or 2 μM PDTC. **D: **NFκB transcriptional activity (as measured by a transiently transfected SEAP reporter vector) in PC12 cells treated for 24 hr with 10 ng/ml NGF, 10 ng/ml TNFα or NGF plus TNFα (Both) in the presence of 0.5 μM L-NAME. Data are shown as mean ± S.E.M. from 3 independent replicate experiments. * and #: p < 0.05 vs. control or TNFα-alone cells, respectively (two-tailed unpaired Student's t-test).

Involvement of NF-κB was further explored by determining the extent to which pharmacological inhibition of NF-κB would block NGF/TNFα-promoted iNOS induction in PC12 cells. As shown in figure 5A, treatment of PC12 cells with either pyrrolidine di-thio-carbamate (PDTC) or the octapeptide proteasome inhibitor PSI (two effective NF-κB inhibitors that have distinct mechanisms of action [8,63-65], completely abolished NGF/ TNFα-promoted iNOS induction. In this experiment, PD98059, a selective MAPK inhibitor, was used as a negative control. Both NF-κB inhibitors effectively blocked NF-κB-mediated transcriptional activity as determined by SEAP reporter gene assay (Figure 5B), whereas PD98059 had no effect. However, PD98059 completely blocked NGF-promoted neurite outgrowth (Figure 5C), an event that in PC12 cells is dependent on MAPK activation [66]. Furthermore, consistent with the results reported in Figure 4, inhibition of NOS activity by L-NAME did not affect NFκB activation by NGF/TNFα combined treatment (Figure 5D).

### NGF/TNFα-promoted iNOS induction requires the simultaneous presence of both the p75NTR and TrkA NGF receptors

**Figure 6:**
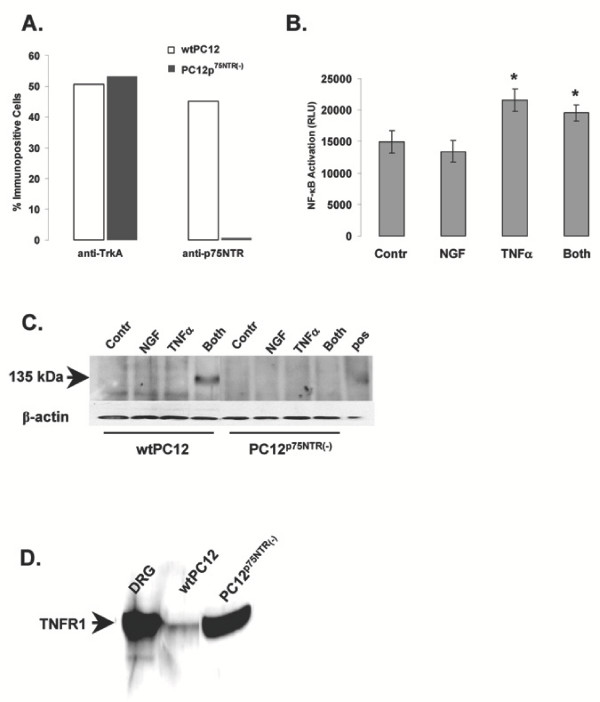
**A: **Graph depicting the percentage of TrkA- or p75NTR- immunopositive cells in wild type (wt)PC12 cells and PC12 cell mutants lacking the low affinity NGF receptor (PC12^p75NTR(-)^) from flow cytometry data. Results shown are representative of 3 replicate flow cytometry experiments on the same cell line. **B: **SEAP release in the culture medium of PC12^p75NTR (-) ^cells transfected for 24 hr with an NF-κB-sensitive SEAP reporter gene construct and treated for 12 hr with vehicle (Cont), 10 ng/ml NGF, 10 ng/ml TNFα or NGF plus TNFα (Both). Data are shown as mean ± S.E.M. from 3 independent replicate experiments. * : p < 0.05 vs. control or NGF-alone cells (two-tailed unpaired Student's t-test). **C: **Western blot detecting the presence of iNOS in wtPC12 cells and PC12^p75NTR (-) ^cells treated for 24 hr with vehicle (Cont), 10 ng/ml NGF, 10 ng/ml TNFα or NGF plus TNFα (Both). Membrane was re-probed for β-actin (lower panel) to control for equal protein loading. Positive control (Pos) is 4 μg of total protein extracts from mouse macrophages. Results shown are representative of 4 replicate experiments. **D: **Western blot detecting the presence of TNFR-I in total protein extracts from wtPC12 cells and PC12^p75NTR (-) ^cells. Twenty μg of total protein extracts from rat dorsal root ganglia (DRG) were used as a positive control.

Next, we subcloned a PC12 mutant cell line (PC12^p75NTR (-)^) that lacks p75NTR expression while retaining TrkA at levels comparable with wild type PC12 cells (Figure 6A). NF-κB activity was not significantly increased by the NGF/TNFα combined treatment over the levels induced by TNFα alone in PC12^p75NTR (-) ^(Figure 6B). Consistent with this finding, PC12^p75NTR (-) ^cells exposed to the combined NGF/TNFα treatment did not show any induction of iNOS expression as compared to the parent cell line (Figure 6C). It is important to note that the PC12^p75NTR (-) ^cells used here express TNFα receptor type 1 (TNFR1) at levels comparable (or even higher) than wild type PC12 cells (Figure 6D). Therefore lack of iNOS induction by the NGF/TNFα combined treatment in these cells cannot be ascribed to lack of TNFα responsiveness (as can also be appreciated by the NFκB response induced by TNFα alone shown in figure 6B).

**Figure 7:**
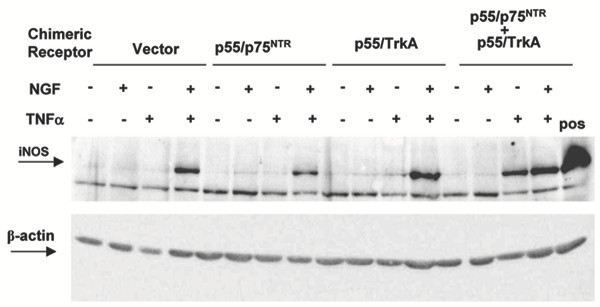
Western blot detecting iNOS in 40 μg total protein extracts from PC12 cells treated for 24 hr with 10 ng/ml human TNFα, 10 ng/ml NGF, or both. Twenty-four hr before treatment, cells were transfected with either an empty vector or expression vectors for chimeric receptor proteins bearing the human TNFR1 ligand binding domains and the intracellular domain of either rat p75^NTR ^or TrkA NGF receptors (p75^NTR^, TrkA or p75NTR+TrkA). Positive control (Pos) is 40 μg of total protein extract from wild type PC12 cells treated with both rat TNFα and NGF. Membrane was re-probed for β-actin (lower panel) to control for equal protein loading and is representative from 3 independent transfections and treatments.

The results obtained in PC12^p75NTR(-) ^would suggest that p75NTR is essential to mediate iNOS induction by the combined TNFα/NGF treatment while the results obtained using K252a (Figure 1) would suggest a prominent role for TrkA. In order to ultimately ascertain the relative role of the two NGF receptors in mediating TNFα/NGF-promoted iNOS induction we made use of PC12 cells transiently transfected with expression vectors coding for chimeric TNFα/NGF receptors constructed as described by Rovelli et al. [77]. These constructs bear the ligand binding domain from the human TNFR1 and the signal transduction domain from rat NGF receptors, either TrkA or p75NTR. Previously, it has been shown that transfection with these chimeras allows for TNF-promoted NGF signaling [77]. Figure 7 shows a western blot detecting iNOS in PC12 cells individually or simultaneously transfected with chimeric TNFα receptors bearing the intracellular domain of p75NTR (p55/p75NTR) or TrkA (p55/TrkA). Transfected cells were then treated either with TNFα and NGF alone, or with both TNFα and NGF. As expected, the combined TNFα/NGF treatment induced a robust expression of iNOS in these PC12 cells, regardless of the presence of any transfected expression vector. As also expected, NGF alone did not elicit iNOS expression in any of the transfected cells. Similarly, TNFα alone did not induce iNOS in cells transfected with either p55/p75NTR or p55/TrkA chimeric receptors. However, TNFα promptly induced iNOS expression in cells transfected with both p55/p75NTR and p55/TrkA chimeric receptors.

## Discussion

The work presented here stems from our original observation that iNOS expression and subsequent NO production can be synergistically induced by NGF and TNFα in a TrkA-dependent manner in PC12 cells [43]. Our present results investigated the signalling pathways involved. Since we consistently observed a higher iNOS expression if NGF is added simultaneously to TNFα, we propose that iNOS expression was induced selectively in NGF-responsive cells. These results do not allow us to rule out the possibility that intermediate factors induced by TNFα or NGF may play a role in sensitizing indirectly cells to NGF or TNFα, respectively. However, the results shown in Figure 2 seem to exclude such a possibility. Indeed, while withdrawal of NGF and/or TNFα allows for a prompt ablation of iNOS expression (Figure 2B), neither NGF nor TNFα alone is sufficient to sustain iNOS expression following withdrawal of TNFα or NGF (Figure 2C). These observations suggest that the simultaneous and continuous presence of both factors is required to sustain iNOS induction/expression and that cell sensitization through a priming mechanism seems unlikely. Nonetheless, other researchers have attributed increased TNFα toxicity in PC12 cells to NGF-induced differentiation [67]. However, our results seem to exclude that differentiation of PC12 cells may have played a role. First, in our experimental conditions iNOS expression occurs as early as 3 hr after the exposure to the combined NGF/TNFα treatment [43], earlier than any morphological differentiation induced by NGF. Second, while blockade of NGF-induced differentiation by the MAPK inhibitor PD98059 (Figure 5C, [68]) had no effect on NGF/TNFα-promoted iNOS expression (Figure 5A), blockade of NFκB did not affect NGF-induced differentiation (Figure 5C) but completely inhibited iNOS expression.

In the present study we also report that induction and maintenance of iNOS expression by the combined NGF/TNFα treatment requires continuous *de novo *iNOS mRNA synthesis, presumably due to transcription factor regulation. Indeed, abolishing iNOS enzymatic activity had no effect on NGF/TNFα-promoted iNOS induction (Figure 4A,B). Therefore, the involvement of positive feedback due to NO seems unlikely. On the other hand, analysis of transcriptional activity of NF-κB, AP-1 and CRE revealed that NF-κB most likely mediates synergistic iNOS induction by TNFα and NGF. Since iNOS induction can be observed as early as 3 hr after NGF/TNFα combined treatment in PC12 cells [43], the results shown in figure 5 suggest that NF-κB is the only transcription factor among those tested here that is responsive to the simultaneous treatment with TNFα and NGF in a fashion consistent with induction of iNOS expression. In fact, while TNFα alone induced NFκB at 3 hr, this induction was significantly lower than the one promoted by the combined NGF/TNFα treatment. Whether the extent to which NFκB is activated or whether qualitative differences in NFκB subunit composition in response to TNFα as compared to NGF/TNFα treatment may play a role in inducing iNOS expression remains to be established. Nonetheless, inhibition of NF-κB completely inhibited iNOS induction while inhibition of MAPK was ineffective (Figure 5A). Lastly, inhibition of NOS activity failed to block NGF/TNFα-promoted NFκB activation, thus further supporting the idea that targeting NO may acutely ameliorate associated oxidative stress, but could not represent the most comprehensive approach to achieve a long term correction of these events.

Previous studies indicated that NGF can induce NF-κB by acting through the low affinity p75^NTR ^receptor [70]. Thus, involvement of NF-κB in mediating NGF/TNFα combined effects would suggest a role for p75NTR. Indeed, we found that mutant PC12 cells that lack expression of the p75NTR receptor failed to respond in terms of iNOS expression when simultaneously treated with NGF and TNFα. Consistent with this finding, in PC12 cell mutants lacking p75NTR expression NF-κB activity was not induced by the combined NGF/TNFα treatment above the levels observed in cells treated with TNFα alone (Figure 6B).

That PC12 cells bearing only the TrkA receptor failed to respond the combined NGF/TNFα treatment suggests that signaling from p75NTR in combination with TNFα is necessary to induce iNOS expression. On the other hand, our previous work illustrated the importance of TrkA-associated signaling in mediating NGF/TNFα-promoted induction of iNOS [43] (see also figure 1). These results are only apparently in contrast. Indeed, in an admittedly artificial system making use of chimeric constructs we observed that only in the presence of both TNFα-responsive NGF receptor signaling can TNFα promote iNOS expression when added alone. Whether this is a consequence of simultaneous but independent signaling of both types of NGF receptors [79] or recruitment of intracellular signalling elements uniquely driven by the simultaneous activation of both NGF receptors' signaling domains remains to be investigated. On the other hand, these results exclude the possibility that the combined action of TNFα and NGF may derive from yet undescribed interaction(s) of the extracellular domains of their respective receptors following ligand binding.

Thus, our combined results would indicate that there exists a specific pathway involving NF-κB and requiring the simultaneous expression or both types of NGF receptors that is synergistically induced by TNFα and NGF to promote expression of iNOS. This is of particular interest given that neuron types expressing both TrkA and p75NTR receptors are limited and known to be affected in neurodegenerative conditions where neuroinflammation and pro-inflammatory cytokines have been shown to play a significant role. Notably, simultaneous expression of TrkA and p75NTR in the CNS is mostly restricted to the BFCN that are known to be particularly affected in AD. Indeed, others have also described signaling pathways that require the simultaneous expression of both TrkA and p75NTR [71,72] as well as the convergence of TrkA and p75NTR-mediated signaling impinging upon NF-κB [73]. Recent reports in neurons of TNF-promoted signaling occurring selectively in the presence of the glutamate agonist NMDA [4] illustrate the importance of considering the signaling "context" when studying the effects of cytokine treatment.

Overall, our data indicate the possibility that a convergence between NGF-promoted trophic signaling and TNFα could selectively endanger NGF-responsive neurons under conditions of neuroinflammation because of a synergistic action between TNFα and NGF to induce iNOS expression. For example, TNFα overexpressing transgenic mice show selective neurodegeneration of NGF-responsive basal forebrain cholinergic neurons [57] and direct TNFα administration in the brain of mice results in an impairment of basal forebrain cholinergic function [58]. However, whether induction of iNOS and subsequent oxidative damage may play a role in these two models remains to be determined [80].

## Conclusion

TNFα and NGF, via concerted signaling events involving NFκB transcriptional activity and targeting NGF-responsive cells bearing both the high and low affinity NGF receptors, converge to stimulate *de novo *transcription of iNOS. Our present results are relevant to neurodegenerative conditions such as AD [22,74], stroke [17,75], ALS [20,76] and spinal chord injury [8,10] where neuroinflammation and high levels of pro-inflammatory cytokines have been shown to play a significant role and proposed as therapeutic targets.

## List of Abbreviations

AraC, cytosine β-D-arabinofuranoside; AD, Alzheimers disease; BDNF, brain derived neurotrophic factor; BFCN, basal forebrain cholinergic neurons; CNS, central nervous system; CRE, cyclic-AMP response element; GDNF, glial derived neurotrophic factor; IGF, insulin-like growth factor; IL-1β, interleukin-1beta; iNOS, inducible nitric oxide synthase; MAPK, mitogen activated protein kinase; NF-κB, nuclear factor kappa B; NGF, nerve growth factor; NO, nitric oxide; nNOS, neuronal nitric oxide synthase; NTR, neurotrophin receptor; PC12, pheochromocytoma; PCN, penicillin; PDTC, pyrrolidinedithyocarbamate; PSI, proteosome inhibitor; SDS, sodium dodecylsulfate; SEAP, secreted alkaline phosphatase; S.E.M, standard error of the mean; Strep, streptomycin; TNFα, tumor necrosis factor alpha; TrkA, troponin-like receptor kinase A; TTBS, tris-buffered saline with tween 20;

## Competing interests

The author(s) declare they have no competing interests.

## Authors' contributions

MST participated in the conception and design of the study, carried out the bulk of experiments, performed data analysis, and drafted the manuscript. PMJ participated in study design especially with regards to the IGF experiments. WZ participated in study design and coordination and provided the expertise for RTPCR and withdrawal experiments. HUS sub-cloned the PC12^p75NTR(-) ^cells and participated in study design and result interpretation of experiments involving these cells. GT participated in conception, study design, coordination and helped to draft and review the manuscript. All authors read and approved the final manuscript.
